# Diffuse large B-cell lymphoma presenting with neurolymphomatosis and intravascular lymphoma: a unique autopsy case with diverse neurological symptoms

**DOI:** 10.1186/1746-1596-7-94

**Published:** 2012-08-13

**Authors:** Sohsuke Yamada, Akihide Tanimoto, Atsunori Nabeshima, Takashi Tasaki, Ke-Yong Wang, Shohei Kitada, Hirotsugu Noguchi, Yasuyuki Sasaguri

**Affiliations:** 1 Department of Pathology and Cell Biology, School of Medicine, University of Occupational and Environmental Health, 1-1 Iseigaoka, Yahatanishi-ku, Kitakyushu, 807-8555, Japan; 2 Department of Molecular and Cellular Pathology, Kagoshima University Graduate School of Medical and Dental Sciences, Kagoshima, 890-8544, Japan; 3 Department of Urology, School of Medicine, University of Occupational and Environmental Health, 1,3. Kitakyushu City, Japan. 2, Kagoshima City, Japan

**Keywords:** Neurolymphomatosis (NL), Peripheral nerve, Intravascular lymphoma (IVL), Diffuse large B-cell lymphoma (DLBL)

## Abstract

A 78-year-old Japanese male noticed a difficulty in the beginning of standing up, followed by 7a progressive numbness of extremities with pain, Bell’s palsy, dysarthria, and difficulty in swallowing. A clinician had suspected cancer of unknown primary origin, accompanied by the diverse and elusive neurological symptoms, likely presenting as painful mononeuropathy simplex and cranial neuropathy. He developed dysbasia over weeks and died 1 month after the symptom onset. At autopsy, an ill-defined large and soft tumor mass in the right lobe of the liver with direct invasion into the right adrenal gland was observed. The left adrenal gland or right iliopsoas muscle was also involved. Microscopic findings showed a monotonous proliferation of medium-sized to large atypical lymphoid cells, which were diffusely positive for CD20 in immunohistochemistry, consistent with diffuse large B-cell lymphoma (DLBL). Furthermore, the lymphoma cells aggressively infiltrated endoneurial and subperineurial spaces not only in the peripheral nerves and plexuses, but partly in the spinal nerve roots, and intravascular spaces in various tissues. Therefore, systemic lymphoma (DLBL) complicated with neurolymphomatosis (NL) and intravascular lymphoma (IVL) was diagnosed. Very early diagnosis and treatment are necessary for the NL patients with poor prognosis.

**Virtual slides:**

The virtual slides for this article can be found here: http://www.diagnosticpathology.diagnomx.eu/vs/5862472377020448.

## Background

Infiltration of the peripheral nerves by lymphoma cells was termed neurolymphomatosis (NL). In 1907, Marek described a disease of Orpington roosters [1], in which the peripheral nerves were infiltrated by lymphocytes, that was tentatively proposed the term NL by Pappenheimer *et al.*, later [2]. Although there had been debated about the propriety of this term [3,4], NL is currently defined as a demonstration of lymphomatous infiltration of the peripheral nerves, representing a unique subtype of extranodal lymphoma [4], and is the least common neurological manifestation of lymphoma. From the comprehensive study by Barron *et al.*[5], between 8.5% and 29% of non-Hodgkin lymphomas, nearly always B-cell lymphomas infiltrated the nervous systems and an estimated 10% of those involved the peripheral nervous system (PNS) on the dignosis of NL. Additionally, approximately one-half cases had widespread systemic lymphoma found at the time of autopsy and 5% had central nervous system (CNS) involvement [4,6]. By contrast, the involvement of a single peripheral nerve or plexus at the only site of malignant lymphoma in NL patients rarely has been reported, and the majority of them has coexistent multiple sites involvement [7-10], including spinal nerve roots (48%), cranial nerves (46%), and plexuses (40%), as more recently described [10]. NL must be separable from more common lymphoma-related neurologic manifestations, such as compression of peripheral nerves by an enlarged lymph node or soft tissue mass, or lymphoma-related non-tumor disorders, e.g., irradiation, chemotherapy, paraneoplastic syndrome, antibody-mediated nerve damage or vasculitis [4,5,8,11]. On the other hand, intravascular lymphoma (IVL) is also considered to be a rare type of extranodal large B-cell lymphoma, characterized by the proliferation of atypical lymphoid cells in the lumina of small vessels in various organs without tendency to form tumors [12]. Recently, it has been suggested that IVL might have a predilection not only for the vessels but for both the CNS and PNS, i.e., the complication with NL [13].

We reported a rare and unique autopsy case of malignant lymphoma complicated with NL and IVL, which clinically presented as progressive painful mononeuropathy multiplex and cranial neuropathy.

## Case presentation

**Figure 1:**
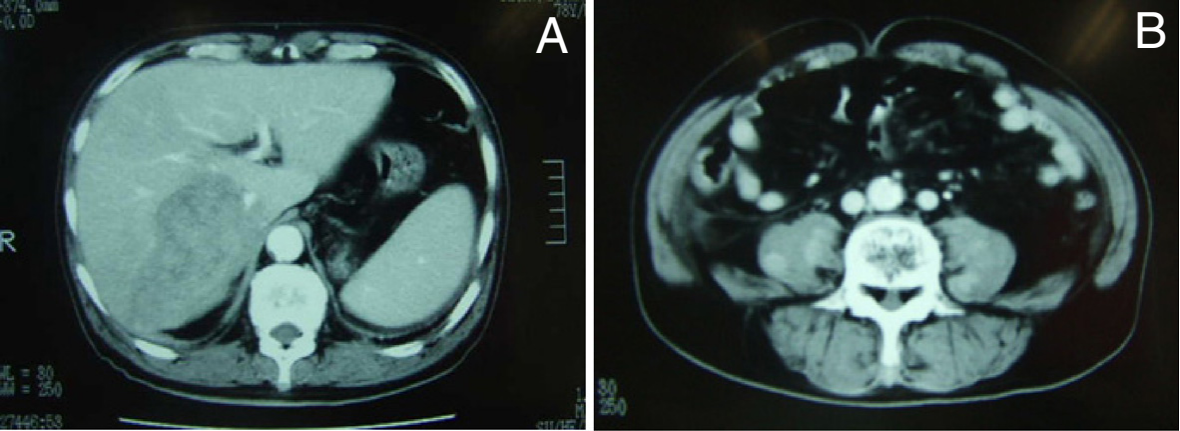
**The findings of an abdominal CT scan. (A)** An ill-defined hepatic mass approximately 9 cm in diameter occupied the right lobe. **(B)** Multiple enhanced nodules in the bilateral iliopsoas muscles were noted.

A 78-year-old Japanese male had 16-year-history of diabetes mellitus with retinopathy and early gastric cancer one year before his death. The gastric cancer was histologically diagnosed as well differentiated tubular adenocarcinoma with no recurrence after the treatment. He noticed a worsening difficulty in the beginning of standing up, followed by a severe and progressive numbness of left upper and both lower extremities, left-sided Bell’s palsy, pain of right lower leg, dysarthria, and swallowing difficulty. The neurological examination including electromyogram revealed mononeuropathy multiplex and cranial neuropathy. The abdominal CT scanning showed an ill-defined and low-density mass, measured approximately 9 cm in diameter, in the right lobe of the liver (Figure1A), coexisted with bilateral adrenal masses and multiple nodules in the right iliopsoas muscle (Figure1B). However, an extensive examination, such as MRI on head or cervical and lumbar vertebrae, revealed no specific findings for explaining the diverse neurological clinical signs. PET scanning had not been examined. Laboratory data, including blood cell counts and biochemistry, were almost within normal limits, except for modestly high levels of lactate dehydrogenase (LDH; 338 IU/L), alkaline phosphatase (ALP; 361 IU/L), and C-reactive protein (CRP; 1.98 mg/dL). Carbohydrate antigen (CA) 19–9 level as a tumor marker was only increased up (54.6 U/mL), but serum interleukin 2 receptor (sIL-2R) was not examined. He developed walking disability over weeks with no response to a steroid pulse therapy (methylprednisolone 1,000 mg/day). The white blood cell count (WBC) showed marked hyperleukocytosis (244,000 /mm^3^) with a differential of 89% neutrophils, 5% lymphocytes (approximately 1% atypical lymphocytes), 5% monocytes, and 1% eosinophils. He died due to a complication of disseminated intravascular coagulation merely 1 month after the symptom onset. The clinician highly suspected cancer of unknown primary origin, associated with multiple metastases of the liver, bilateral adrenal glands, and iliopsoas muscle. There was no history of immunosuppressive disorders, use of immunosuppressive medications, or unusual infections.

### Pathological findings

At autopsy, the liver, weighing 1,540 g, was involved by an ill-defined whitish soft mass in the right lobe (S5-S6), together with direct invasion into the right adrenal gland (80 g). The enlarged left adrenal gland (100 g) and right iliopsoas muscle were also involved. Multiple and relatively small nodular tumor lesions, in left lower lobe of the lung, pericardium, and lymph nodes of colonic serosa, mesenterium and para-pancreas were recognized. In addition, a splenomegaly was noted, weighing 350 g. The brain could not be examined due to the family’s objections.

**Figure 2:**
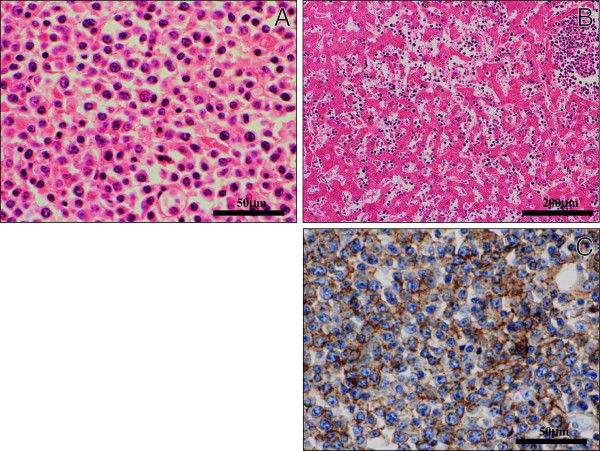
**Histological and immunohistochemical examination of DLBL. (A)** On high-power view, these tumor lesions were composed of a diffuse and monotonous proliferation of large to medium-sized atypical lymphoid cells having enlarged hyperchromatic nuclei and prominent nucleoli. (H&E stains, Original magnification × 400, Bar = 50 μm) (**B**) In the liver, not only nodular growth pattern but sinusoidal proliferation of the lymphoma cells were evident. (H&E stains, Original magnification × 100, Bar = 200 μm) (**C**) Immunohistochemically, these atypical cells were diffusely positive for CD20. (Original magnification × 400, Bar = 50 μm).

Histologically, all these multiple lesions were composed of a diffuse and monotonous proliferation of large to medium-sized atypical lymphocytic cells having hyperchromatic pleomorphic nuclei and prominent nucleoli (Figure2A), which systemically infiltrated multiple organs, including the spleen, bilateral kidney and adrenal glands, pancreas, bone marrow, heart and salivary glands. In the liver, not only nodular (i.e., local) growth pattern but sinusoidal (i.e., systemic) proliferation of the lymphoma cells were evident (Figure2B). In immunohistochemistry, these cells were diffusely positive for CD45 (Dako Cytomation Co., Tokyo, Japan, diluted 1:200) and CD20 (Dako, diluted 1:200) (Figure2C), and very weakly positive for CD79a (Dako, diluted 1:50), whereas negative for CD5 (NOVOCASTRA laboratories Ltd., Newcastle, United Kingdom, diluted 1:25), CD10 (NOVOCASTRA, diluted 1:20), CD30 (Dako, diluted 1:40), bcl-2 (Dako, diluted 1:30), CD3 (Dako, diluted 1:1), CD45RO (UCHL-1; Dako, diluted 1:200), Kappa (Dako, diluted 1:6,000), Lambda (Dako, diluted 1:8,000), and myeloperoxidase (MPO; Dako, dilutede 1:300). All immunohistochemical stainings were carried out using Dako Envision kit (Dako) according to the manufacturer’s instructions. Based on these features, we made a diagnosis of systemic non-Hodgkin lymphoma, diffuse large B-cell lymphoma (DLBL).

**Figure 3:**
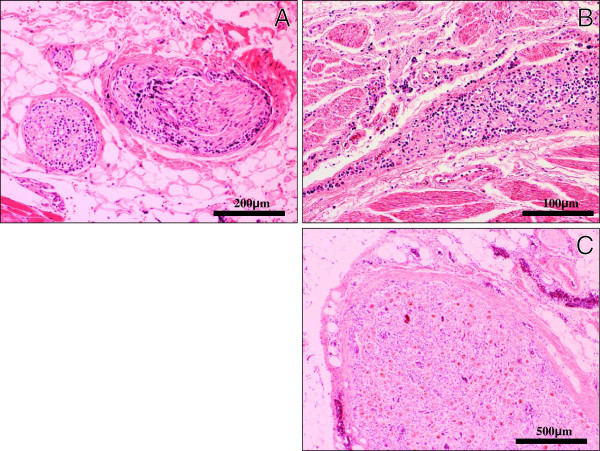
**Histological examination of NL. (A)(B)(C)** On low- to high-power views, the lymphoma cells diffusely infiltrated the endoneurium and subperineurium of not only the peripheral nerves (**A**) and plexuses (**B**), but the lumbar spinal nerve roots (**C**). (H&E stains, Original magnification: A; × 100 B; × 40, C; × 200, Bar: A; = 200 μm, B; = 500 μm, C; = 100 μm).

**Figure 4:**
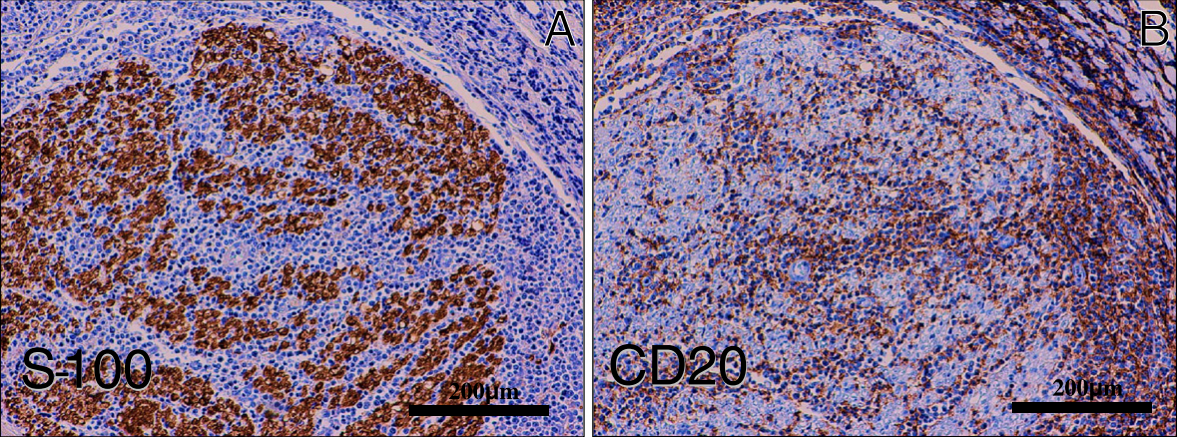
**Immunohistochemical examination of NL. (A)(B)** Immunohistochemical S-100 protein (**A**) and CD20 (**B**) stainings could make it much easier to understand the microscopic findings of NL in the PNS of iliopsoas muscle. (Original magnification × 100, Bar = 200 μm).

**Figure 5:**
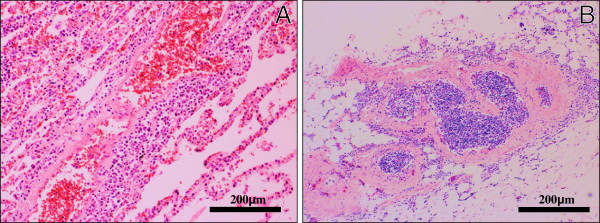
**Histological examination of IVL.** On high-power view, a systemically diffuse intravascular spreading of the lymphoma cells was observed in the alveolar septum (**A**) and the small vessels of iliopsoas muscle (**B**), suggestive of coexisting IVL. (H&E stains, Original magnification × 100, Bar = 200 μm).

Furthermore, the lymphoma cells diffusely infiltrated the endoneurium and subperineurium of not only the peripheral nerves and plexuses in the examined various tissues (Figure3A-B), but the lumbar spinal nerve roots (Figure3C). While, the spinal cord revealed no remarkable change, carrying no apparent lymphoma cells. These findings were consistent with widespread systemic lymphoma (DLBL) complicated with neurolymphomatosis (NL), even while we could never examine the histological findings in the cranial nerves and the sensory PNS of the limb. Immunohistochemical CD20 (Dako, diluted 1:200) and S-100 protein (Dako, diluted 1:900) stainings could make it much easier to understand the microscopic findings of NL in the PNS (Figure4A-B). Also, the intravascular spreading of the lymphoma cells was observed in the alveolar septum (Figure5A), hepatic and splenic sinuses, or small vessels of the various organs (Figure5B). Based on the above clinical and pathological findings, it was suggested that this case was really a coexisting IVL, rather than a leukemic transformation of a DLBL.

## Discussion

NL is characterized by lymphomatous infiltration of PNS. From the first report of the large series of NL by Diaz-Arrastia *et al.*[4], the most common clinical presentation was progressive sensorimotor neuropathy, although less common syndromes including Guillain-Barre, focal peroneal or sciatic neuropathy, a relapsing neuropathy, cauda equina syndrome and multiple cranial neuropathy were described. Therefore, clinicians should consider several differential diagnoses, such as leptomeningeal lymphomatosis, nerve damage from herpes zoster, chemoradiotherapy, nerve root compression, lymphoma-associated vasculitis, or paraneoplastic syndromes [14]. The patterns of clinical presentations in NL are categorized into four: 1) painful involvement of nerves or roots, 2) cranial neuropathy with or without pain, 3) painless involvement of peripheral nerves and 4) painful or painless involvement of a single peripheral nerve.^5^ The present case showed complicated and mixed type of these four characteristics. In contrast, IVL is also known to show the diversity of non-specific clinical symptoms, including fever elevation, respiratory distress, and neurologic abnormalities, e.g., sensory and motor deficits or altered consciousness [14]. In the current case, it is very difficult to make it clear to distinguish IVL-induced neurological signs from NL-induced ones, since detailed gross and microscopic examinations have never been performed within limited specimens obtained. Additionally, most of previous studies have not addressed PNS involvement in patients with IVL, however, Matsue *et al.*, based on the collection of 4 IVL patients complicated with NL, have described that it is very likely that IVL has a predilection not only for the vascular vessels but for the PNS [14]. Despite the extreme rarity of IVL, DLBL complicated with IVL and NL may be more common than generally considered.

Odabasi *et al.* reported a review of 52 NL cases that approximately one-half of them had also widespread systemic lymphoma found at the time of autopsy [6]. The diagnosis in many cases had been difficult, since a multidisciplinary approach for obtaining an adequate biopsy specimen of the suspected nerve was required for clinicians. Moreover, it is well known that the prognosis is very poor due to the progressive characteristics, as shown in the present case. When NL occurs in the context of established malignant lymphoma, the main cause of death is disseminated disease with associated multiple organ failure, but respiratory failure secondary to severe neuropathy might also occur [11]. Thus, early and accurate diagnosis and aggressive treatment (e.g., high-dose chemotherapy and/or radiation therapy) can increase their survival rates [4,5,9,11,14]. MRI would be essential and helpful to diagnose, because it can detect the number of the NL lesions and the extent of NL involvement. From the previous report by Amar Swarnkar *et al.*[15], MRI showed mild persistent enlargement, hyperintensity on T2-weighted images and enhancement after gadolinium administration in the involved nerves. However, these radiographic findings are nonspecific and nondiagnostic, very similar to our case. More recently, PET scanning is considered to be a useful diagnostic tool to evaluate the distribution of involvement for patients with suspected NL, but is even unlikely to be 100% positive and at least one report of a false-negative case has been published [14]. After all, nerve biopsies are required to obtain an accurate and correct diagnosis of NL. Approximately 83% of nerve biopsies are reported to show lymphomatous infiltration especially in the endoneurium, less commonly in the subperineurium [4-6]. Therefore, suspicion of NL needs a multidisciplinary approach because a biopsy would be performed on the most likely both clinically and radiologically suspected nerve. Consultation with surgeons, who experienced in peripheral nerve procedures including fascicular nerve biopsy, should be recommended [11].

## Conclusion

We herein reported an autopsy case of DLBL strongly associated with pathological findings of NL and IVL in the clinical background of diverse neurological symptoms. Although NL and/or IVL are uncommon features, we pathologists should be aware that, in particular, the NL patients undergo a fatal condition with very poor prognosis, and that the diagnosis of NL requires an accurate and correct approach for an adequate biopsy specimen of the suspected nerves.

## Consent

Written informed consent was obtained from the patient for publication of this case report and any accompanying images. A copy of the written consent is available for review by the Editor-in-Chief of this journal.

## Competing interests

The authors declare that they have no competing interests.

## Authors’ contributions

SY and AT participated in conception of the idea and writing of the manuscript. SY, AT, AN, TT, KYW, SK, HN and YS performed the histological interpretation of the tumor tissue. All authors have read and approved the final manuscript.
